# Vibration Characteristics of FG Shaft–Double-Disk Rotor System for Power Turbines

**DOI:** 10.3390/ma19173731

**Published:** 2026-09-02

**Authors:** Tao Liu, Haochen Hu, Ping Xiang, Qingshan Wang

**Affiliations:** 1 School of Civil Engineering, Central South University, Changsha 410075, China; liutaosr@csu.edu.cn (T.L.);; 2 College of Mechanical and Electrical Engineering, Central South University, Changsha 410083, China; 3 State Key Laboratory of Precision Manufacturing for Extreme Service Performance, Central South University, Changsha 410083, China

**Keywords:** shaft–double-disk system, rotor dynamics, functionally graded materials, energy method, free vibration analysis

## Abstract

This study establishes a dynamic model for a functionally graded (FG) shaft–double-disk rotor system under rotational motion. The effective properties of the functionally graded material (FGM) are described using the Voigt homogenization scheme. Within the frameworks of first-order shear deformation theory and Timoshenko beam theory, the Lagrangian energy functional of the system is derived using the energy method, and the free vibration characteristics are obtained through a variational solution based on the Ritz method. The accuracy of the proposed method is validated through comparisons with published results and finite element method (FEM) simulations, with a maximum deviation of 0.7808% from the benchmark solutions. The parametric results show that increasing the shaft inner-to-outer radius ratio increases the natural frequencies, whereas increasing the power-law index, disk dimensions, or disk spacing generally reduces the natural frequencies and critical rotational speeds. Among the investigated parameters, increasing the disk radius from 50 to 150 mm reduces the third natural frequency by approximately 49.3%, while increasing it from 60 to 120 mm reduces the critical rotational speed by up to approximately 29.7%. These findings provide quantitative guidance for the dynamic design of FG power-turbine rotor systems.

## 1. Introduction

As a highly efficient and flexible power device, the gas turbine plays a central role in today’s industrial sector, with irreplaceable importance in key areas such as energy, transportation, national defense, and industrial drive systems. The power turbine, which is specifically responsible for delivering effective mechanical output in a gas turbine, necessitates an in-depth investigation of its dynamic behavior. Since the power turbine is often modeled as a shaft–disk rotor system in most engineering analyses, studying the dynamic characteristics of such systems serves as a critical reference for power turbine design. Currently, power turbines are developing toward a higher efficiency and reduced weight. Nevertheless, these components often function in extreme environments characterized by elevated temperatures and pressures, placing rigorous demands on material capabilities. As a result, there is an increasing need to employ advanced composite materials with an enhanced performance in rotor assemblies, especially in critical applications such as power turbines. Hence, developing and evaluating a dynamic model for an FG shaft–double-disk rotor system is of substantial engineering importance, providing critical insights for the practical design and production of power turbines.

As a class of advanced nonhomogeneous composite materials, FGMs exhibit continuously varying material properties in space. The multifunctionality of FGM is achieved by adjusting the spatial distribution of constituent components. Traditional composite materials often suffer from issues such as interfacial delamination during service; FGMs alleviate these problems by weakening the interlayer interfaces, thereby improving the overall structural integrity. Recent studies have extensively explored the dynamic behavior of the FG plate and shell structures, with significant contributions from various researchers. Ebrahimi et al. [1,2,3] established the spatially discretized equations for FG circular plates under classical boundary conditions using variable transformation, exploring their free vibration behavior under general boundary conditions. Employing the first-order shear deformation theory (FSDT) as the theoretical foundation, Jomehzadeh et al. [4] established an analytical dynamic model for functionally graded sectorial plates and conducted a comprehensive investigation into their stress characteristics. In their pioneering work, Hashemi et al. [5,6] developed new closed-form analytical approaches to explicitly derive the governing equations describing the free vibration of functionally graded circular and annular plates, with a further examination of the dynamic behavior exhibited by rotating FG plate configurations. Through exact analytical approaches, Saidi et al. [7,8] systematically examined both the vibrational response and buckling performance of moderately thick functionally graded sector plates mounted on elastic foundation systems. Aghdam et al. [9] employed an integrated approach combining the Kantorovich method with FSDT to investigate the static behavior of functionally graded sector plates. Within the framework of classical plate theory, Fereidoon et al. [10] conducted a rigorous analytical–numerical study of the flexural and vibrational response of functionally graded annular sector plates subject to classical boundary constraints. Their implementation of the Kantorovich solution technique was systematically verified through extensive numerical demonstrations of the method convergence and solution accuracy. Building upon three-dimensional elasticity theory, Dong [11] formulated a comprehensive dynamic model to analyze functionally graded annular plates subjected to classical boundary conditions. The investigation employed the Ritz variational approach to derive solutions, ultimately characterizing the plate’s free vibration behavior through rigorous analytical treatment.

The shaft–disk rotor system, composed of shafts and disks, is commonly modeled as a combination of beam and annular plate structures in dynamic analyses. As beams and annular plates are also fundamental structural elements in engineering, they have been the subject of extensive research and investigation. Considering the influence of Coriolis forces on rotating structures, Lin et al. [12] combined the principles of virtual work and D’Alembert’s principle to establish the discretized vibration equations for rotating Timoshenko beams. They solved for the natural frequencies under rotation and investigated the effects of the angular velocity and slenderness ratio on the vibration characteristics of the structure. Grounding their work in the fundamental principle of virtual displacements and Navier’s analytical approach, Giunta et al. [13] conducted a comprehensive vibration analysis of simply supported beams. By implementing both classical and higher-order theoretical frameworks, they rigorously evaluated the effects of the slenderness ratio and material properties on the beam’s dynamic response. Wang et al. [14] utilized the differential quadrature method to formulate a dynamic model of multi-walled carbon nanotube beams within the framework of the Timoshenko beam theory, investigating the dependence of natural frequencies on the slenderness ratio. By synthesizing a newly developed beam theory with Hamilton’s variational principle, Sina et al. [15] derived a comprehensive free vibration formulation for functionally graded beams under generalized boundary conditions. The study systematically investigated how boundary constraints and material property variations influence the spectral characteristics and modal configurations of the beam system. Based on a three-degree-of-freedom shear deformation theoretical framework, Aydogdu [16] constructed a vibration analysis model for generally constrained laminated composite beams. The proposed methodology’s accuracy was rigorously verified via extensive comparative numerical studies with established solutions. Utilizing wave propagation principles, Xu et al. [17] established a comprehensive dynamic formulation for beams with fractional-order viscoelastic properties. Their methodology enabled a complete characterization of the structural vibrational behavior, encompassing both free oscillation modes and forced vibration regimes. Ding et al. [18] formulated the spatially discretized equations for laminated annular plates based on three-dimensional elasticity theory and solved the free vibration responses under various boundary conditions using the finite Hankel transform method. Through rigorous three-dimensional elasticity theory, Xu [19] established an exact solution framework to comprehensively analyze the vibration characteristics of laminated annular plates, accounting for both classical simply supported and non-classical elastically constrained sliding boundary conditions. Alipour et al. [20] combined the principle of minimum potential energy with the double-superimposed zigzag theory to construct the stress–strain theoretical model for FG sandwich annular plates under non-uniform traction loads. Numerical examples demonstrated both the high accuracy and significantly improved computational efficiency of the method. Hashemi et al. [21] formulated a three-dimensional dynamic model for functionally graded annular plates with clamped boundaries using elasticity theory, employing the Ritz method to determine their free vibration characteristics. Extending this work, Tajeddini et al. [22] introduced a Polynomial-Ritz numerical approach to analyze free vibration in variable-thickness FG annular plates under arbitrary boundary conditions and elastic foundation support. Their results confirmed the excellent convergence and high computational accuracy. Utilizing the Ritz variational approach, Su et al. [23] conducted a comprehensive free vibration analysis of functionally graded annular plates. Their study quantitatively assessed the effects of different boundary constraint types and material property gradations on the plate’s dynamic response characteristics.

In shaft–disk rotor systems, the coupled vibration characteristics between the shaft and the disk have become a central research focus and have attracted extensive scholarly attention in recent years. Through a comparative implementation of three different theoretical frameworks, Chiu et al. [24] established comprehensive dynamic models for shaft–disk rotor systems. Their work systematically characterized the coupled vibrational behavior and quantified the influence of disk compliance on overall system dynamics. Building upon this foundation, Liu et al. [25] proposed an assumed-mode-based finite element formulation for stepped shaft–disk configurations, with particular emphasis on elucidating how disk flexibility modifies the spectral properties of higher-order natural frequencies. Combining Kirchhoff plate theory for disk modeling with Euler–Bernoulli beam theory for shaft representation, Heydari et al. [26] developed a dynamic formulation for shaft–disk systems using the assumed mode method. Their study systematically examined how variations in the flexible disk positioning and geometric aspect ratios modify the system’s free vibrational response. Tuzzi et al. [27] investigated the coupled vibration responses of shaft–disk rotor systems and discussed the driving mechanisms of internal coupling effects within the rotor. Eshelman et al. [28] constructed a dynamic model of shaft–disk systems under the influence of gyroscopic torque and analyzed how gyroscopic moments affect the critical rotational speed of the rotor system. Zhou et al. [29] analyzed the coupled vibration characteristics of shaft–disk rotor systems using the assumed mode method and FEM, exploring the effects of disk flexibility and tie rod positions on the system’s free vibration response. She et al. [30] established a continuous model for shaft–disk rotor systems and studied the internal coupled vibration behavior, particularly focusing on the influence of the rotational speed on the system’s natural frequencies. Entezari et al. [31] used FEM to build a dynamic model of rotor systems and investigated how the disk thickness impacts the dynamic behavior of the rotor. Shahab et al. [32] constructed a dynamic model for shaft–disk rotor systems and examined the influence of shaft flexibility on the system’s dynamic characteristics as well as internal coupling effects within the rotor system.

The existing literature indicates that the current research on rotor systems has primarily focused on homogeneous materials or shaft–single-disk configurations, while studies on FG flexible shaft–double-disk systems remain relatively limited. Therefore, further investigation in this area is necessary to fill the research gap. Although FEM is well-established, reduced-order analytical–numerical formulations can provide a computationally efficient alternative for repeated parametric analyses while retaining satisfactory accuracy. The present work develops a numerical framework for analyzing the free vibration behavior in functionally graded shaft–double-disk rotor systems. By integrating Timoshenko beam theory with FSDT, this study systematically determines the system’s dynamic response characteristics. Comprehensive numerical investigations demonstrate the validity and robustness of the proposed analytical approach. On this basis, the effects of key internal parameters of the rotor system on its free vibration behavior are further explored, providing theoretical insights for the future dynamic analysis and design of power turbine rotor systems.

The main contributions of this work are summarized as follows: First, a free-vibration model is established for a rotating FG shaft–double-disk rotor system by coupling Timoshenko beam theory for the shaft with first-order shear deformation theory for the disks. Second, the governing equations are derived through an energy-based Ritz formulation that explicitly includes the effects of rotational speed and gyroscopic coupling. Third, the proposed method is validated against published solutions and FEM simulations in both stationary and rotating conditions, and the modal agreement is further assessed through a mode-shape comparison. Finally, the effects of the power-law index, shaft geometry, disk dimensions, and disk spacing on the natural frequencies and critical speeds are quantified to provide design-oriented insight into power turbine rotor systems.

## 2. Theoretical Deductions

### 2.1. Geometric Description of the Rotor System

**Figure 1:**
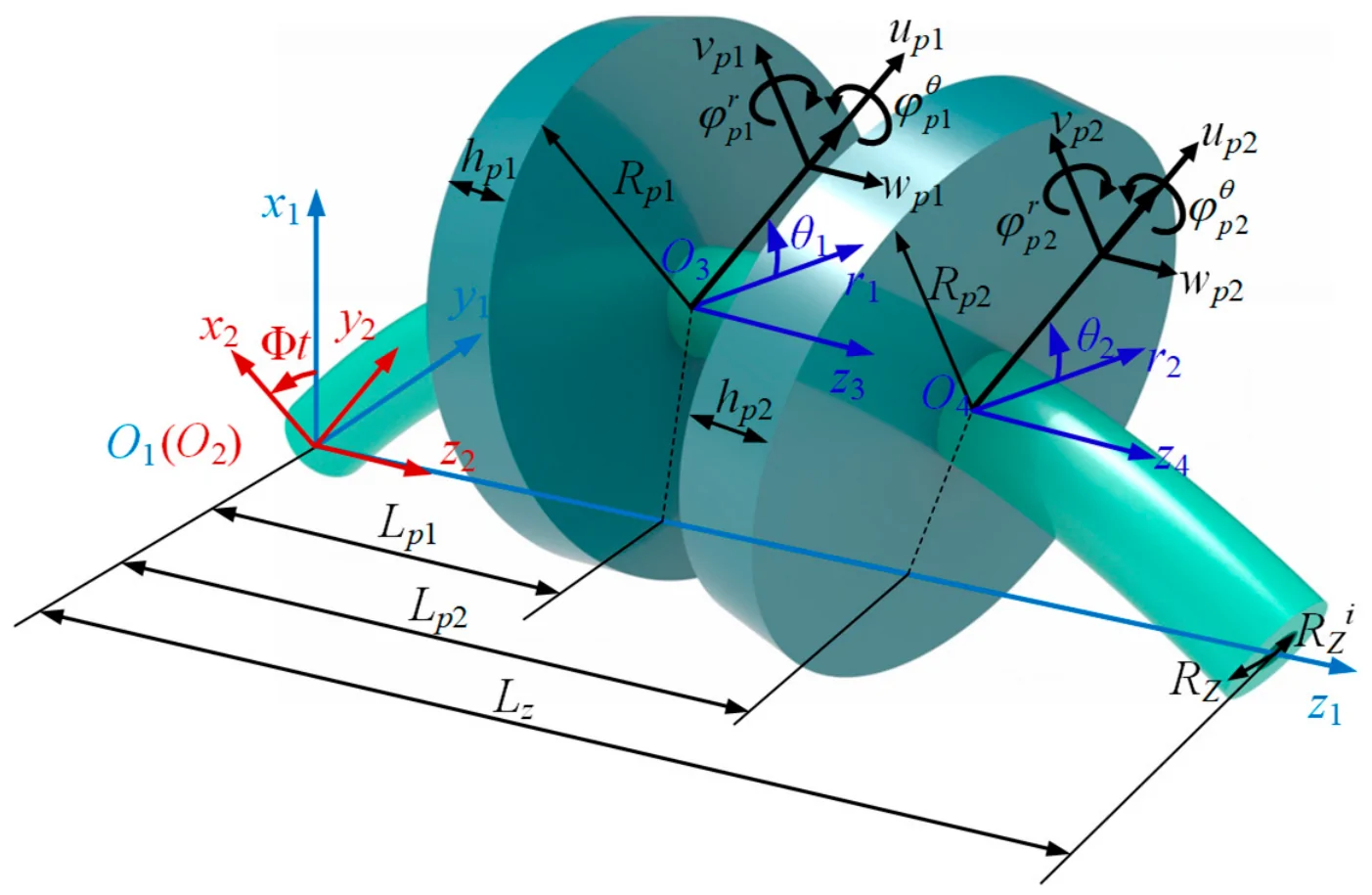
Structural and coordinate system diagram of the shaft–double-disk system.

Figure 1 presents the schematic configuration of the composite rotor system, consisting of a shaft and two functionally graded disks.

To adequately capture both the rigid-body dynamics and elastic deformation in this complex system, four distinct coordinate systems are implemented. These coordinate systems include the global coordinate system *O*_1_-*x*_1_*y*_1_*z*_1_, the rotating coordinate system *O*_2_-*x*_2_*y*_2_*z*_2_, and the oscillating coordinate systems *O*_3_-*r*_1_*θ*_1_*z*_3_ and *O*_4_-*r*_2_*θ*_2_*z*_4_, denoted, respectively, as *D*_1_, *D*_2_, *D_p_*_1_, and *D_p_*_2_. In Figure 1, the coordinate system *D*_1_ originates at point *O*_1_ on the shaft’s left end, with its *z*_1_-axis coinciding with the undeformed shaft centerline. This primary reference frame characterizes the rotor system’s absolute velocity and displacement fields. The origin *O*_2_ of *D*_2_ coincides with *O*_1_, and the *z*_2_-axis aligns with *z*_1_. This coordinate system rotates uniformly with an angular velocity Φ, capturing the deformation of the shaft and the rigid-body displacement at the shaft–disk coupling nodes. The coordinate systems *D*_3_ and *D*_4_ originate at the geometric centers of disks 1 and 2, respectively. Their *z*_3_- and *z*_4_-axes are normal to the corresponding disk midplanes, enabling the characterization of disk elastic deformation. In this rotor system, the shaft has a length *L_z_*, an inner radius $R_{z}^{i}$ and an outer radius *R_z_*. Disks 1 and 2 have thicknesses *h_p_*_1_ and *h_p_*_2_, and radii *R_p_*_1_ and *R_p_*_2_, respectively. The shaft–disk coupling nodes for disks 1 and 2 are located at distances *L_p_*_1_ and *L_p_*_2_, respectively, from the shaft’s left end. While actual rotor systems employ bearing supports, this study models the shaft supports as equivalent elastic springs for analytical simplification.

The unit vectors of *D*_1_, *D*_2_, *D_p_*_1_, and *D_p_*_2_ are denoted as ***d***_1_, ***d***_2_, ***d**_p_*_1_, and ***d**_p_*_2_, respectively. The transformation relationships among these unit vectors are as follows:

(1) $$\left\{\begin{array}{l} d_{2}=H_{\Phi }d_{1}\\ d_{\xi }=H_{\xi }d_{2},\xi =p_{1},p_{2} \end{array}\right.$$

The specific expressions for the transformation matrices **H**_Ω_ and **H***_ξ_* are given as follows:

(2) $$H_{\Phi }=\left[\begin{array}{ccc} 1 & 0 & 0\\ 0 & \cos\Phi t & \sin\Phi t\\ 0 & -\sin\Phi t & \cos\Phi t \end{array}\right]$$

(3) $$H_{\xi }=H_{\xi }^{x}H_{\xi }^{y}H_{\xi }^{z}$$

(4) $$H_{\xi }^{x}=\left[\begin{array}{ccc} 1 & 0 & 0\\ 0 & \cos\theta_{\xi }^{x} & \sin\theta_{\xi }^{x}\\ 0 & -\sin\theta_{\xi }^{x} & \cos\theta_{\xi }^{x} \end{array}\right]$$

(5) $$H_{\xi }^{y}=\left[\begin{array}{ccc} \cos\theta_{\xi }^{y} & 0 & -\sin\theta_{\xi }^{y}\\ 0 & 1 & 0\\ \sin\theta_{\xi }^{y} & 0 & \cos\theta_{\xi }^{y} \end{array}\right]$$

(6) $$H_{\xi }^{z}=\left[\begin{array}{ccc} \cos\theta_{\xi }^{z} & \sin\theta_{\xi }^{z} & 0\\ -\sin\theta_{\xi }^{z} & \cos\theta_{\xi }^{z} & 0\\ 0 & 0 & 1 \end{array}\right]$$

### 2.2. Dynamic Modeling and Solving of Rotor Systems

Following Timoshenko beam theory, the shaft’s bending deformation is characterized by translational displacements (*u_z_*, *v_z_*) and rotational displacements ($\theta_{z}^{x}$, $\theta_{z}^{y}$). In coordinate system D1, any point on the shaft cross-section can be located as follows:

(7) $$d_{z}=\left(d_{O_{2}O_{\xi }}+d_{O_{\xi }z}H\right)H_{\Phi }$$

(8) $$d_{O_{2}O_{\xi }}=\left[\begin{array}{ccc} u_{z} & v_{z} & z_{z} \end{array}\right]$$

(9) $$d_{O_{\xi }z}=\left[\begin{array}{ccc} r\cos\theta & r\sin\theta & 0 \end{array}\right]$$

where the coordinate *z_z_* represents the axial location of the cross-section within reference frame *D*_2_. Based on Timoshenko beam kinematics, the kinetic energy functional of the rotating shaft is derived as follows:

(10) $$T_{z}=\frac{1}{2}\int_{0}^{L_{z}}\int_{0}^{2\pi }\int_{R_{z}^{i}}^{R_{z}}\rho {\dot{d}}_{z}^{2}rdrd\theta dz$$

As illustrated in Figure 1, the geometric centers of disks 1 and 2 are rigidly coupled to the shaft’s neutral axis, enforcing the orthogonality between each disk’s inner boundary plane and the shaft’s *z*_2_-axis at the attachment locations. For an arbitrary material point on either rotating disk, the spatial position can be mathematically described by the following:

(11) $$d_{p}=\left(d_{O_{2}O_{\xi }}+d_{O_{\xi }p}H\right)H_{\Phi }$$

(12) $$d_{O_{2}O_{\xi }}=\left[\begin{array}{ccc} u_{z} & v_{z} & z_{z} \end{array}\right]$$

(13) $$d_{O_{\xi }p}=\left[\begin{array}{ccc} r\cos\theta +u_{p}^{a}\cos\theta -v_{p}^{a}\sin\theta & r\sin\theta +u_{p}^{a}\sin\theta +v_{p}^{a}\cos\theta & z_{z}+w_{p}^{a} \end{array}\right]$$

where $u_{p}^{a}$, $v_{p}^{a}$, and $w_{p}^{a}$ represent the *x*-, *y*-, and *z*-directional displacement components, respectively, for any material point on the disk. Within the framework of FSDT, these displacement fields are kinematically related to the generalized displacement vector $d_{p}^{a}$ = [*u_p_*, *v_p_*, *w_p_*, $\theta_{p}^{r}$, $\theta_{p}^{\theta }$]^T^ through the following transformation [33,34]:

(14) $$\left[\begin{array}{c} u_{p}^{a}\\ v_{p}^{a}\\ w_{p}^{a} \end{array}\right]=\left[\begin{array}{ccccc} 1 & 0 & 0 & 0 & z\\ 0 & 1 & 0 & z & 0\\ 0 & 0 & 1 & 0 & 0 \end{array}\right]d_{p}^{a}$$

The expression for the kinetic energy of the disk is given by the following [35]:

(15) $$T_{\xi }=\frac{1}{2}\int_{-\frac{h_{\xi }}{2}}^{\frac{h_{\xi }}{2}}\int_{0}^{2\pi }\int_{R_{z}}^{R_{\xi }}\rho_{\xi }{\left({\dot{d}}_{p}^{a}\right)}^{2}rdrd\theta dz$$

The kinetic energy of the disk consists of three components: the kinetic energy due to the disk’s own elastic vibrations, the kinetic energy induced by the shaft’s vibration, and the energy associated with the internal coupling effects within the system.

Following Timoshenko beam theory, the shaft’s strain–displacement relationships are expressed as follows:

(16) $$\varepsilon_{z}=\left[\begin{array}{cccc} 0 & 0 & -yE_{01}^{\left(1\right)} & 0\\ 0 & 0 & 0 & -xE_{01}^{\left(1\right)}\\ E_{01}^{\left(1\right)} & 0 & -X & 0\\ 0 & E_{01}^{\left(1\right)} & 0 & -X \end{array}\right]\tau_{z}$$

where the strain vector of the shaft is **ε***_z_*= [$\varepsilon_{z}^{x}$, $\varepsilon_{z}^{y}$, $\varepsilon_{z}^{xz}$, $\varepsilon_{z}^{yz}$]^T^, the displacement vector is **τ***_z_*= [*u_z_*, *v_z_*, $\theta_{z}^{x}$, $\theta_{z}^{y}$]^T^, **X** is the identity matrix, and $E_{01}^{\left(1\right)}$ is the first-order weighting coefficient matrix. The expression for the shaft stress is as follows:

(17) $$\nu_{z}=\left[\begin{array}{cccc} X_{z} & & & \\ & X_{z} & & \\ & & \lambda Y_{z} & \\ & & & \lambda Y_{z} \end{array}\right]\varepsilon_{z}$$

Here, **X***_z_* and **Y***_z_* are *N_z_*-dimensional diagonal matrices, and the shear coefficient is given by *λ* = 6(1 + *μ*)^2^/(7 + 12*μ* + 4*μ*^2^), where *μ* is the Poisson’s ratio. The expression for the strain energy of the shaft is as follows [36]:

(18) $$V_{z}=\frac{1}{2}\int_{0}^{L_{z}}\int_{0}^{2\pi }\int_{R_{z}^{i}}^{R_{z}}\nu_{z}^{T}\varepsilon_{z}drd\theta dz$$

The expression for the spring potential energy of the supporting springs at both ends of the shaft is as follows:

(19) $$V_{z}^{s}=\frac{1}{2}\left(d_{z}^{T}k_{z}^{1}d_{z}{|}_{z=0}+d_{z}^{T}k_{z}^{2}d_{z}{|}_{z=L_{z}}\right)$$

where $k_{v}^{\Lambda }$ = diag($k_{u}^{1}$, $k_{v}^{1}$, $k_{w}^{1}$) (Λ = 1, 2) represents the stiffness coefficients of the supporting springs at both ends of the shaft.

The strain vector of the disk **ε***_p_*= [$\varepsilon_{r}^{p}$, $\varepsilon_{\theta }^{p}$, $\gamma_{r\theta }^{p}$, $\chi_{r}^{p}$, $\chi_{\theta }^{p}$, $\chi_{r\theta }^{p}$, $\gamma_{rz}^{p}$, $\gamma_{\theta z}^{p}$]^T^ can be expressed in terms of the displacement components as follows:

(20) $$\varepsilon_{p}=\frac{1}{r}\left[\begin{array}{ccccc} rE_{02}^{\left(1\right)} & 0 & 0 & 0 & 0\\ X & F_{02}^{\left(1\right)} & 0 & 0 & 0\\ F_{02}^{\left(1\right)} & rE_{02}^{\left(1\right)}-X & 0 & 0 & 0\\ 0 & 0 & 0 & rE_{02}^{\left(1\right)} & 0\\ 0 & 0 & 0 & X & F_{02}^{\left(1\right)}\\ 0 & 0 & 0 & F_{02}^{\left(1\right)} & rE_{02}^{\left(1\right)}-X\\ 0 & 0 & rE_{02}^{\left(1\right)} & r & 0\\ 0 & 0 & F_{02}^{\left(1\right)} & 0 & r \end{array}\right]d_{p}^{a}$$

where $E_{02}^{\left(1\right)}$ and $F_{02}^{\left(1\right)}$ are second-order weighting coefficient matrices. According to Hooke’s law, the disk stress vector ν*_p_* = [$N_{p}^{r}$, $N_{p}^{\theta }$, $N_{p}^{r\theta }$, $M_{p}^{r}$, $M_{p}^{\theta }$, $M_{p}^{r\theta }$, $Q_{p}^{r}$, $Q_{p}^{\theta }$]^T^ relates to the strain vector ε*_p_* through constitutive relations, as detailed in Ref. [37]. The strain energy expression for the disk is as follows:

(21) $$V_{\xi }=\frac{1}{2}\int_{R_{z}}^{R_{\xi }}\int_{0}^{2\pi }\nu_{p}^{T}\varepsilon_{p}rd\theta dr$$

In this study, the energy method is employed to solve for the dynamic characteristics of the structure. The energy functional expression of the rotor system is given by the following:

(22) $$G=T_{z}+T_{p1}+T_{p2}-V_{z}-V_{p1}-V_{p2}-V_{z}^{s}$$

By applying the Rayleigh–Ritz method to the energy functional of the rotor system, the variational solution yields the spatially discretized dynamic equation of the rotor system:

(23) $$M\ddot{\Theta }+G\dot{\Theta }+K\Theta =0$$

where **M**, **G**, and **K**, respectively, denote the system’s mass, gyroscopic, and stiffness matrices, while **Θ** represents the vector of generalized displacement coefficients.

### 2.3. Description of Material Models

This study considers an isotropic shaft coupled with FG disks. The FGM is an inhomogeneous composite that combines multiple material phases with spatially varying compositions. Following the Voigt model, the effective material properties are determined by constituent properties *T* and volume fractions *V* as follows:

(24) $$T=T_{1}V_{1}+T_{2}V_{2}$$

where subscripts “1” and “2” represent two different constituent materials, and their volume fractions satisfy the relation *V*_1_ + *V*_2_ = 1.

The volume fraction *V*_1_ of the primary constituent follows a power-law distribution through the disk thickness, expressed as follows:

(25) $$V_{1}={\left(z^{a}+\frac{1}{2}\right)}^{p},z^{a}=\frac{z}{h_{\xi }}$$

where *p* is the power-law index, taking values in the range [0,+∞), and the thickness coordinate *z^a^* satisfies −0.5 ≤ *z^a^* ≤ 0.5. When *p* = 0, the FGM consists entirely of material 1, whereas it gradually approaches material 2 as *p* → ∞.

The Voigt homogenization scheme is adopted here because it provides a direct and computationally efficient means of describing the continuous variation in the effective material properties according to the prescribed volume fractions of the ceramic and metal constituents. Its simple formulation can be readily combined with the above power-law distribution and incorporated into the present energy-based Ritz framework. This treatment is consistent with the objective of the present study, which focuses on the effects of the material gradation and structural parameters on the global vibration characteristics of the rotor system rather than on detailed material microstructures.

Nevertheless, the Voigt model assumes perfect bonding and an iso-strain condition between the constituent phases. It does not explicitly account for the constituent morphology, spatial distribution, phase interactions, or local stress concentrations. In particular, the predicted effective elastic stiffness generally represents an upper-bound estimate and may therefore produce a relatively stiff response compared with more elaborate homogenization approaches. Alternative schemes, such as the Mori–Tanaka or self-consistent approaches, may be considered in future investigations focusing on detailed microstructural effects.

## 3. Numerical Results and Analysis

Section 2 established a theoretical model for the free vibration analysis of a shaft–FG disk rotor system. This section validates the model’s effectiveness, and investigates how the structural dimensions, material parameters, and rotational speed influence the vibration characteristics. Unless otherwise specified, the geometric parameters are $R_{z}^{i}$ = 0.006 m, *R_z_* = 0.018 m, *L_z_* = 0.57 m, *h_p_*_1_ = *h_p_*_2_ = 0.014 m, *R_p_*_1_ = *R_p_*_2_ = 0.12 m, *L_p_*_1_ = 0.45 m, and *L_p_*_2_ = 0.51 m. The material properties of the FGM constituents are listed in Table 1, and simply supported boundary conditions are applied to the shaft unless otherwise specified.

### 3.1. Validity Analysis

In this section, the effectiveness of the proposed method is validated through comparisons with published results and FEM simulations. To quantitatively evaluate the agreement between the different solutions, the absolute relative error of each natural frequency is calculated as Error (%) = (*f*_Reference_ − *f*_Present_)/*f*_Reference_ × 100%, where *f*_Present_ denotes the natural frequency obtained using the proposed method, while *f*_Reference_ denotes the corresponding frequency reported in the literature or obtained from FEM, depending on the comparison considered. Table 2 compares the natural frequencies of the FG shaft–disk system obtained using the proposed method with those reported in Ref. [38]. The material properties and structural dimensions are taken from the cited study. The results reveal a maximum deviation of 0.7808%, demonstrating that the method introduced in this study is both accurate and dependable for analyzing the free vibration behavior of FGM rotor systems.

It should be noted that the modal numbering in Table 2 follows that adopted in Ref. [38] to enable a direct one-to-one comparison. Because the benchmark system exhibits paired symmetric modes, only the representative modes reported in the reference are included, while their symmetric counterparts are not repeated in the table.

**Figure 2:**
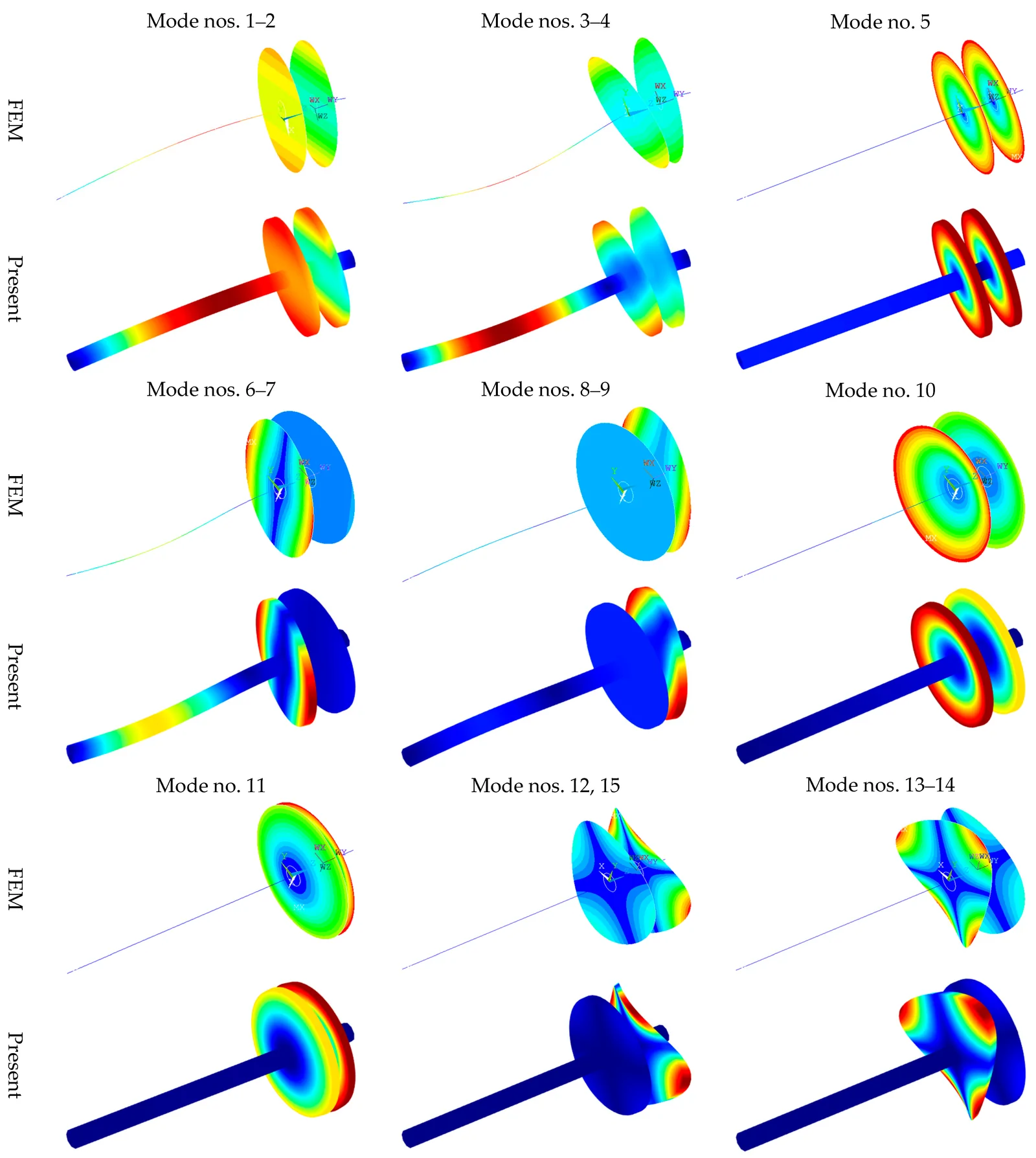
Comparison of the first 15 orders of modes of the shaft–double disks system.

Table 3 and Figure 2 compare the natural frequencies and mode shapes of the homogeneous shaft–double-disk system obtained using the proposed method and FEM. Table 3 provides a quantitative comparison of the first nine natural frequencies, whereas Figure 2 extends the mode-shape comparison to the first fifteen modes to further examine the agreement at higher modal orders. The structural dimensions and material parameters used are as follows: $R_{z}^{i}$ = 0.006 m, *R_z_* = 0.018 m, *L_z_* = 0.57 m, *h_p_*_1_ = *h_p_*_2_ = 0.014 m, *R_p_*_1_ = *R_p_*_2_ = 0.12 m, *L_p_*_1_ = 0.45 m, *L_p_*_2_ = 0.51 m, *E* = 210 GPa, *ρ* = 7850 kg/m^3^, and *μ* = 0.3. For this comparison, the relative errors reported in Table 3 were calculated according to the definition given above, with the corresponding FEM frequency taken as *f*_Reference_. The natural frequencies and mode shapes obtained using the proposed method are in close agreement with the FEM results, confirming the accuracy of the proposed method for the free-vibration analysis of the stationary shaft–double-disk system.

The computational efficiency of the two methods was also compared for the validation case presented in Table 3. Under the same computational conditions, the FEM calculation required 348 s, whereas the present method required only 18.86 s. The present method therefore reduced the computational time by approximately 94.58% and was approximately 18.45 times faster than FEM for this case. Combined with the close agreement in the predicted natural frequencies and mode shapes, this result demonstrates that the proposed method can substantially reduce the computational cost while maintaining satisfactory accuracy. This advantage is particularly beneficial for the repeated eigenvalue calculations involved in the subsequent parametric analyses.

After confirming the proposed method’s effectiveness in evaluating the free vibration behaviors of the rotor system at rest, this section proceeds to validate its accuracy in calculating the natural frequencies of the shaft–double-disk system during rotation. Table 4 compares the natural frequencies obtained from the proposed method with those derived via FEM at different rotational speeds. The structural dimensions and material properties employed remain the same as those provided in Table 3. The relative errors in Table 4 were calculated using the corresponding FEM frequencies as the reference values. The close agreement obtained at different rotational speeds further demonstrates the applicability and accuracy of the proposed method for the vibration analysis of the rotating shaft–double-disk system.

Overall, the comparisons with the published results and FEM simulations presented in Table 2, Table 3 and Table 4 and Figure 2 confirm the accuracy and applicability of the proposed method for predicting the natural frequencies and mode shapes of the rotor system under both stationary and rotating conditions.

### 3.2. Parametric Analysis

**Figure 3:**
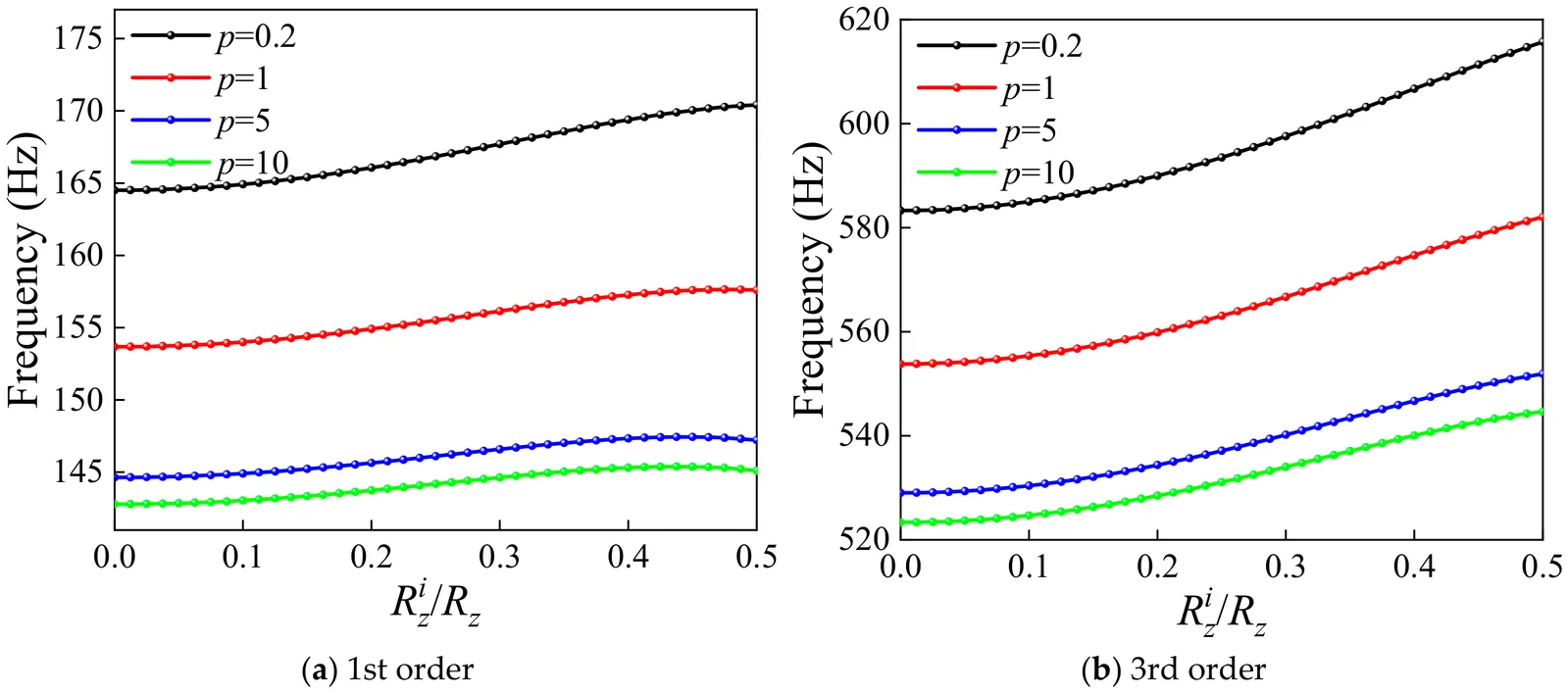
Variation of the natural frequencies of the system with shaft radius under.

**Figure 4:**
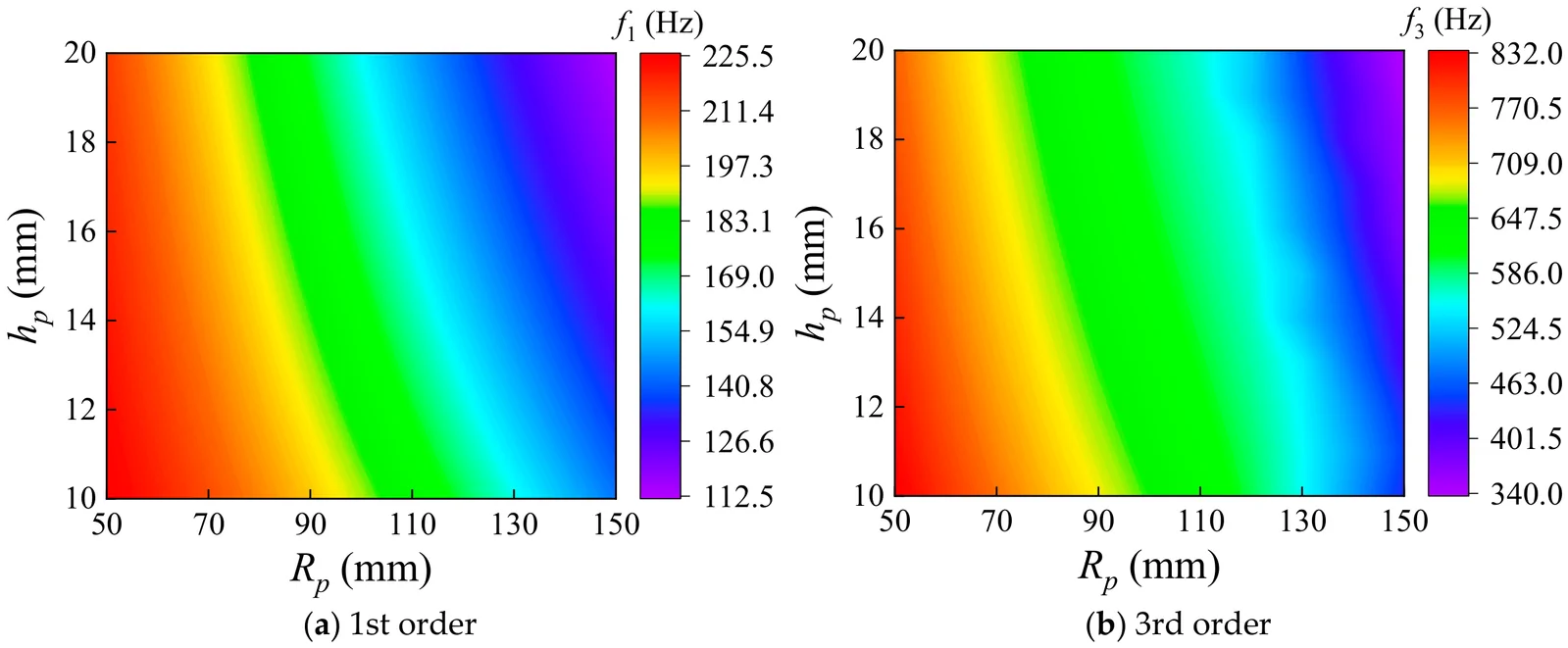
Variation of the natural frequencies of the system with the dimensions of the disks.

**Figure 5:**
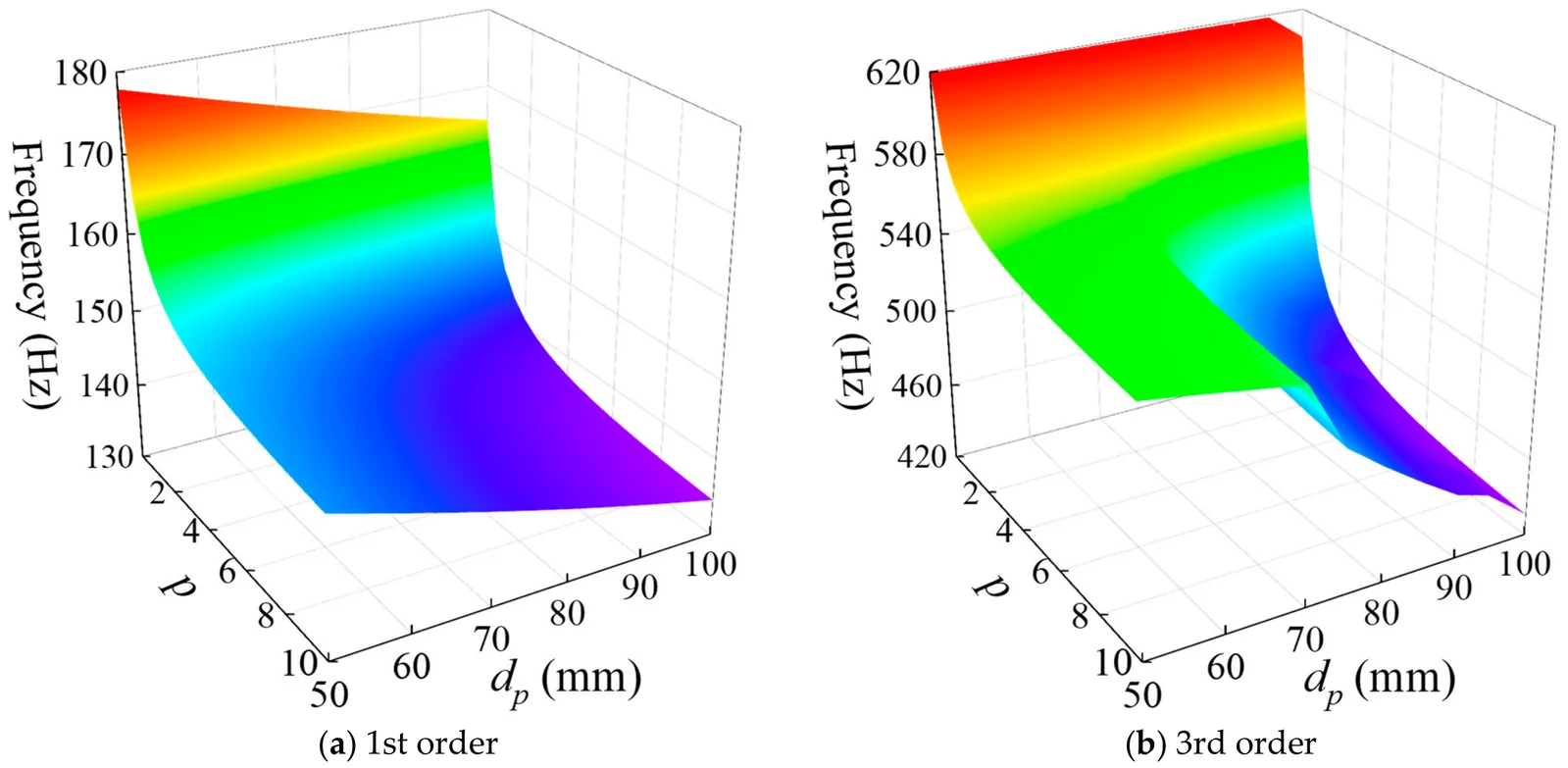
Variation of the natural frequencies of the system with *d_p_* and the power-law index.

**Figure 6:**
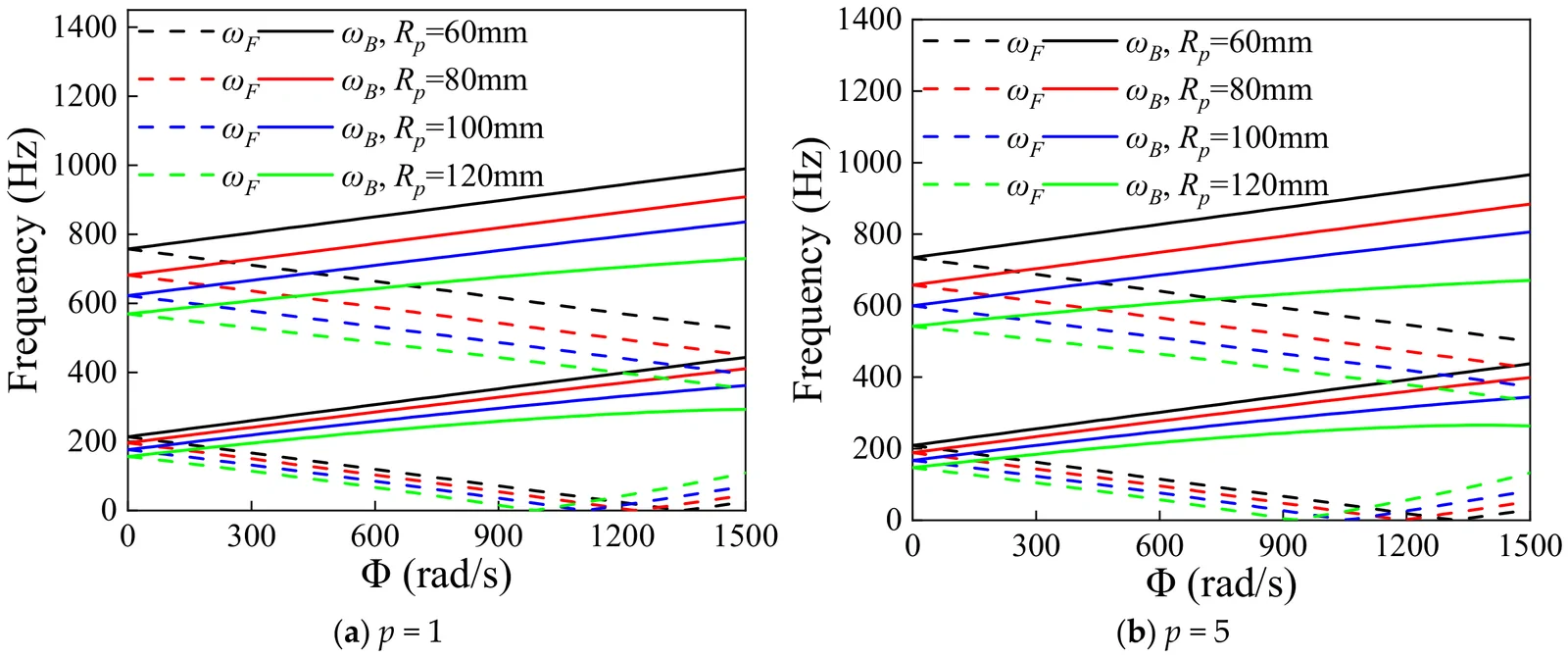
Campbell diagram of the system for different values of *R_p_*.

**Figure 7:**
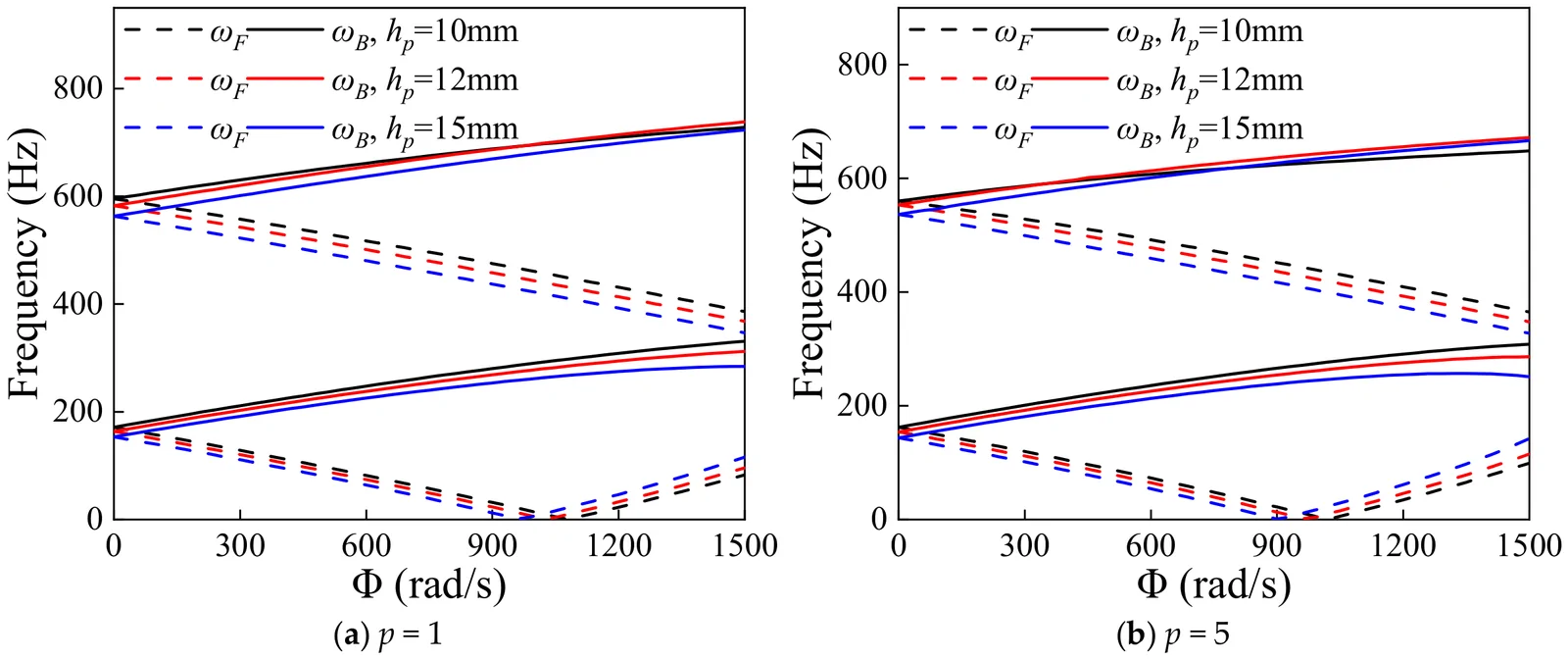
Campbell diagram of the system for different values of *h_p_*.

**Figure 8:**
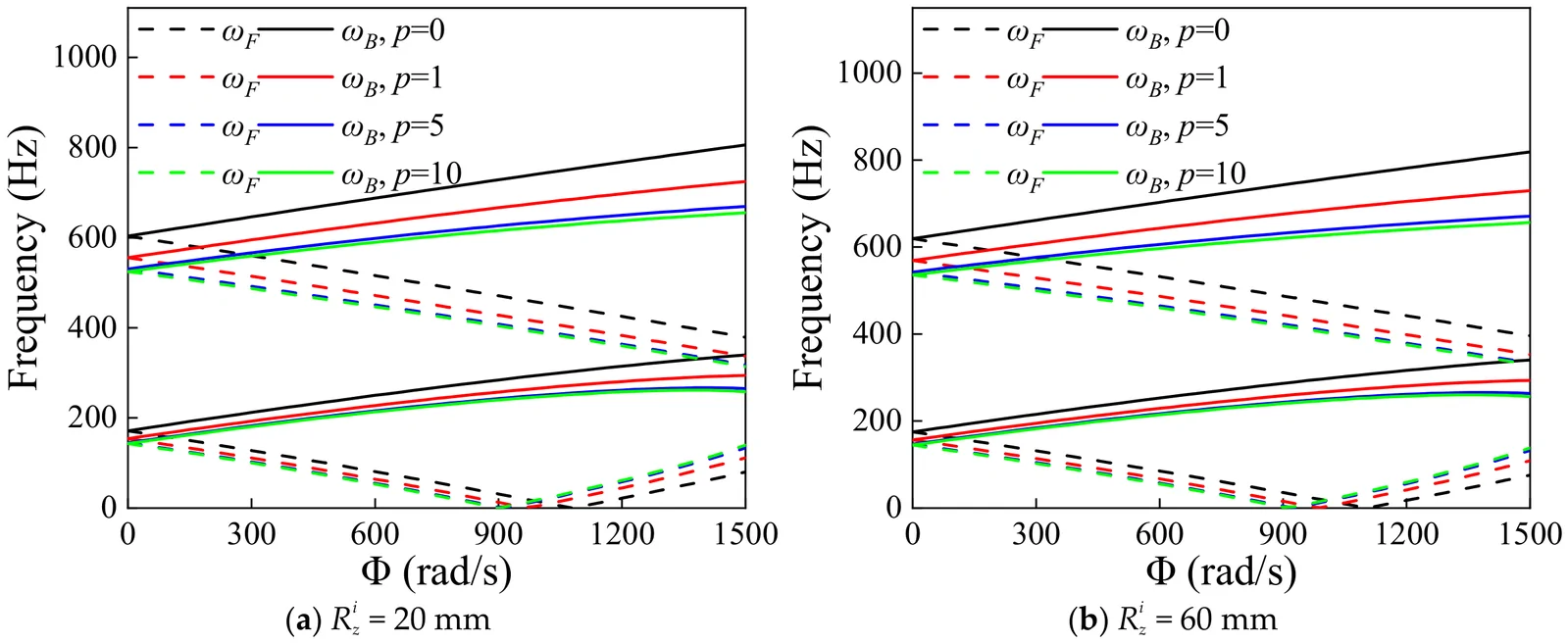
Campbell diagram of the system for different power-law indices.

**Figure 9:**
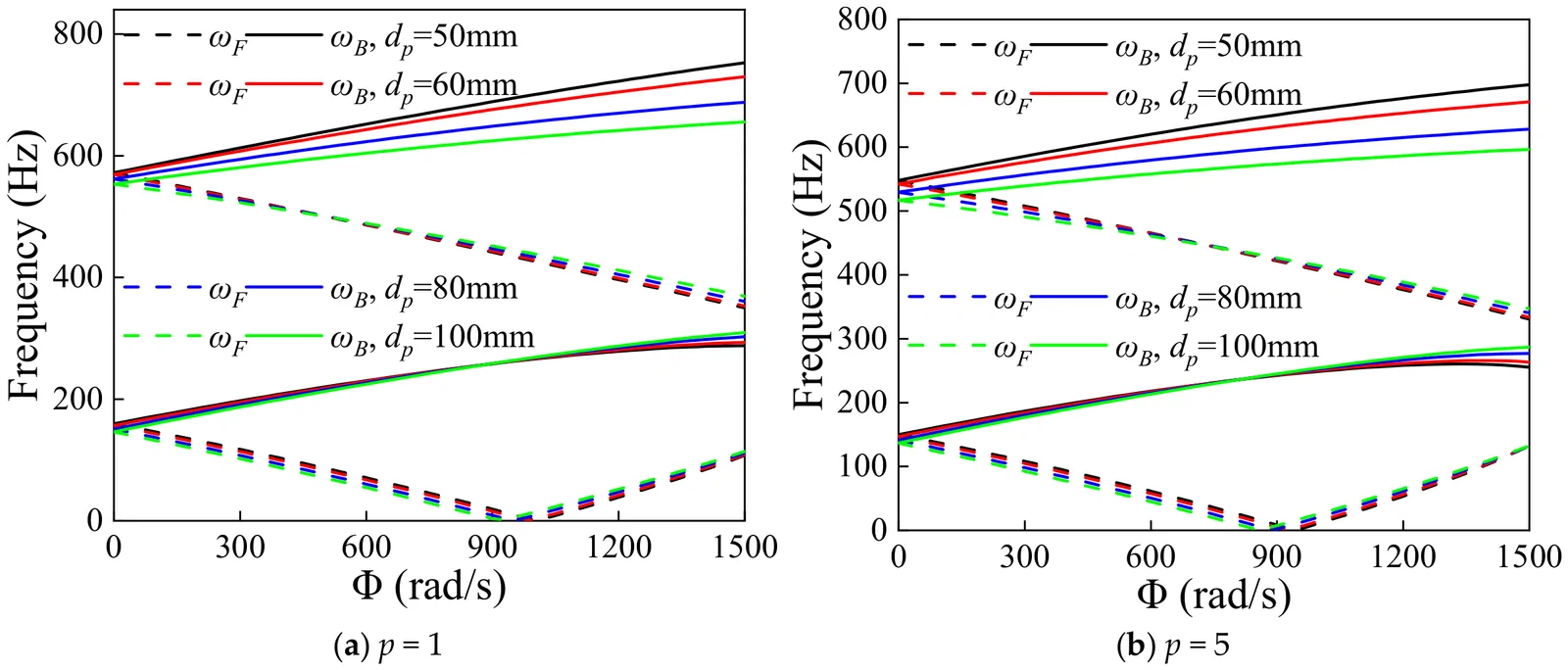
Campbell diagram of the system for different values of *d_p_*.

Following a series of numerical examples that confirmed the effectiveness of the proposed method in evaluating the free vibration characteristics of the shaft–double-disk system under both stationary and rotating states, this section extends the analysis to investigate the free vibration behavior of the system under various conditions. The influence of *p*, the shaft cross-sectional geometry, the disk dimensions, and the spacing between the two disks on the vibration behavior of the FG rotor system is examined. Unless otherwise specified, the power-law index in the examples of this section is set to *p* = 1. It is also worth noting that Figure 3, Figure 4 and Figure 5 investigate the free vibration characteristics of the shaft–double-disk system under stationary conditions, while Figure 6, Figure 7, Figure 8 and Figure 9 examine those under rotating conditions.

Figure 3 illustrates how the natural frequencies of the system vary with changes in the shaft’s cross-sectional dimensions under different power-law indices. In this case, the outer radius of the shaft *R_z_* = 18 mm is kept constant, and the inner radius $R_{z}^{i}$ is varied to change the shaft’s cross-sectional size. The results indicate that, for different power-law indices, the natural frequencies of the shaft–double-disk system increase with the rise in the ratio $R_{z}^{i}$/*R_z_*. It is also observed that, as the power-law index *p* increases, the natural frequencies of the FG shaft–double-disk system tend to decrease. Within a certain range, although the bending stiffness and mass of the system both decrease with increasing $R_{z}^{i}$/*R_z_*, the rate of decrease in the bending stiffness is smaller than that of the mass, causing their ratio to increase, thereby enhancing the system’s natural frequency. As *p* increases, the FG disks tend to be more metal-like. Since metals have relatively lower elastic moduli and densities compared to ceramics, their natural frequencies are generally lower. Consequently, the natural frequency of the system decreases as *p* increases.

Figure 4 illustrates the effects of the disk radius *R_p_* and thickness *h_p_* on the natural frequencies of the stationary shaft–double-disk system. The natural frequencies decrease monotonically as either parameter increases, although the magnitude of the reduction depends on the modal order. Increasing the disk dimensions increases the mass and rotational inertia contributed by the flexible disks. Although the disk stiffness also increases with thickness, the increase in the overall inertia of the coupled rotor system is more influential within the investigated range, resulting in lower natural frequencies. The disk radius has a particularly pronounced effect because the associated increase in the mass moment of inertia becomes increasingly significant as the material is distributed farther from the rotation axis. For example, when *h_p_* = 14 mm, increasing *R_p_* from 50 to 150 mm decreases the third natural frequency by approximately 49.3%. By comparison, when *R_p_* = 120 mm, increasing *h_p_* from 10 to 20 mm decreases the first natural frequency by approximately 18.3%. These results indicate that the disk radius is an important geometric parameter governing the vibration characteristics of the coupled rotor system.

Figure 5 shows the effects of the power-law index *p* and the spacing *d_p_* between the two disks on the natural frequencies of the stationary rotor system. The natural frequencies decrease as either *p* or *d_p_* increases. An increase in *p* changes the FG disks from a ceramic-rich composition toward a more metal-rich composition, thereby modifying the effective stiffness-to-mass relationship of the disks and reducing the frequencies of the coupled system. At *d_p_* = 60 mm, increasing *p* from 0 to 10 decreases the first natural frequency by approximately 17.3%. Increasing the disk spacing also extends the effective deformation span between the two disks and increases their influence on shaft bending, leading to a reduction in the effective bending stiffness of the coupled system. At *p* = 1, increasing *d_p_* from 50 to 100 mm decreases the third natural frequency by approximately 14.4%. The results therefore show that both the material gradation and disk arrangement can be used to adjust the modal characteristics of the rotor system, with their effects varying among the different modal orders.

During rotation, the centrifugal effect modifies the effective dynamic characteristics of the shaft–double-disk system, while the Coriolis-related terms introduce gyroscopic coupling. At zero rotational speed, the corresponding forward- and backward-whirl components have the same frequency. As the rotational speed increases, the gyroscopic coupling breaks this frequency degeneracy and causes the two branches to separate. According to the whirl-frequency notation adopted in this study, *ω_B_* increases monotonically with rotational speed, whereas *ω_F_* initially decreases to zero and subsequently increases. The increasing separation between these branches reflects the strengthening gyroscopic influence as the rotational speed increases. The splitting of the forward- and backward-whirl frequencies changes their intersections with the synchronous excitation line and therefore affects the critical rotational speeds of the rotor system. These intersections indicate the operating speeds at which synchronous resonance may occur. Against this physical background, Figure 6 presents the Campbell diagrams of the system for different values of *R_p_*. As *R_p_* increases, both *ω_F_* and *ω_B_* decrease, and the corresponding critical rotational speed shifts to a lower value. This behavior can be attributed to the reduction in the effective stiffness-to-inertia ratio caused by the increase in disk radius.

Figure 7 presents the Campbell diagrams of the shaft–double-disk system for different disk thicknesses *h_p_*. For each value of *h_p_*, the forward and backward whirl branches gradually separate as the rotational speed increases because of the increasing gyroscopic effect. Increasing *h_p_* shifts both frequency branches toward lower values and moves the intersection between the forward whirl branch and the synchronous excitation line to a lower rotational speed. This behavior indicates that the increase in disk mass and rotational inertia has a greater influence on the system dynamics than the associated increase in disk stiffness within the investigated range. When *p* = 5, increasing *h_p_* from 10 to 15 mm reduces the critical rotational speed by approximately 11.5%. Therefore, a thicker disk causes the rotor system to reach its critical condition at a lower operating speed.

Figure 8 illustrates the variations in the forward whirl frequency, backward whirl frequency, and critical rotational speed for *p* = 0, 1, 5, and 10. The characteristic frequency splitting remains evident for all material distributions. As *p* increases, both whirl-frequency branches shift downward, and their intersection with the synchronous excitation line occurs at a lower rotational speed. This trend results from the transition of the FG disks from a ceramic-rich composition toward a more metal-rich composition, which reduces the effective stiffness-to-inertia relationship of the coupled system. For $R_{z}^{i}$ = 0.006 m, increasing *p* from 0 to 10 decreases the critical rotational speed by approximately 17.7%. The material gradation of the disks therefore has a noticeable influence on the dynamic stability range of the rotor system.

Figure 9 shows the Campbell diagrams obtained for different values of the spacing *d_p_* between the two disks. The forward and backward whirl branches exhibit similar overall shapes for all the considered disk positions, but both branches move slightly toward lower frequencies as *d_p_* increases. Correspondingly, the intersection defining the critical rotational speed also shifts toward a lower value. Increasing the disk spacing enlarges the effective deformation span and enhances the influence of the disks on shaft bending, thereby reducing the effective bending stiffness of the coupled rotor system. Nevertheless, the variation is less pronounced than those caused by the disk dimensions and material gradation. At *p* = 5, increasing *d_p_* from 50 to 100 mm reduces the critical rotational speed by approximately 8.6%. This relatively moderate variation indicates that disk spacing provides a secondary means of adjusting the rotating vibration characteristics within the investigated range.

Overall, Figure 3, Figure 4 and Figure 5 demonstrate the effects of the material gradation and structural parameters on the natural frequencies of the stationary rotor system, while the Campbell diagrams in Figure 6, Figure 7, Figure 8 and Figure 9 show how these parameters influence the whirl-frequency branches and critical rotational speeds under rotating conditions.

## 4. Conclusions

An energy-based model has been developed to investigate the free vibration characteristics of a rotating FG shaft–double-disk rotor system. The shaft and flexible disks are described using Timoshenko beam theory and first-order shear deformation theory, respectively, and the governing equations are solved using the Rayleigh–Ritz method. The formulation incorporates rotational and gyroscopic effects and is applicable to both stationary and rotating conditions. Its accuracy is verified through comparisons with published results and FEM simulations, including natural frequencies and mode shapes under stationary conditions and natural frequencies at different rotational speeds. The maximum deviation from the published benchmark results is 0.7808%, confirming the accuracy of the proposed formulation.

For the stationary system, the material gradient and structural parameters have distinct effects on the natural frequencies. Within the investigated ranges, increasing the shaft inner-to-outer radius ratio from 0 to 0.5 increases the third natural frequency by up to approximately 5.6% at *p* = 0.2. In contrast, increasing the disk radius from 50 to 140 mm at *h_p_* = 14 mm reduces the third natural frequency by approximately 42.4%, representing the most pronounced natural-frequency variation observed in the present parametric study. When *R_p_* = 120 mm, increasing the disk thickness from 10 to 19 mm decreases the first natural frequency by approximately 16.9%. Furthermore, increasing the power-law index from 0 to 9.5 at *d_p_* = 60 mm reduces the first natural frequency by approximately 17.2%, while increasing the disk spacing from 50 to 95 mm at *p* = 1 reduces the third natural frequency by approximately 11.7%. These results show that the decreases in the effective stiffness-to-inertia ratio caused by material and geometric variations generally lead to lower natural frequencies.

For the rotating system, gyroscopic coupling causes the corresponding forward- and backward-whirl frequency branches to separate as the rotational speed increases. This frequency splitting changes the intersections of the whirl-frequency branches associated with the critical rotational speeds of the system. Among the investigated parameters, the disk radius produces the largest critical-speed variation. At *p* = 5, increasing *R_p_* from 60 to 120 mm reduces the critical rotational speed from approximately 1318.3 to 926.5 rad/s, corresponding to a reduction of approximately 29.7%. Increasing the disk thickness from 10 to 15 mm and the power-law index from 0 to 10 reduces the critical rotational speed by up to approximately 11.5% and 17.7%, respectively. By comparison, increasing the disk spacing from 50 to 100 mm produces a smaller maximum reduction of approximately 8.6%. These quantitative findings demonstrate how the material gradient and structural configuration shift the natural frequencies and critical rotational speeds within the parameter ranges considered and provide useful guidance for avoiding resonance in the design of power-turbine rotor systems.

The present formulation is limited to the linear free vibration analysis of the rotor system without considering damping or external excitation. It is therefore primarily applicable to predicting intrinsic dynamic characteristics, including natural frequencies, mode shapes, gyroscopic frequency splitting, and critical rotational speeds, rather than forced-response amplitudes or transient vibration behavior under realistic operating loads. Nevertheless, the current energy-based Ritz framework provides a basis for further developments. Structural, material, and support damping may be incorporated through appropriate damping models, while rotor unbalance and other external excitations may be introduced through their corresponding generalized forces. Such extensions would enable the investigation of synchronous forced responses, resonance amplitudes, and transient vibration characteristics under more realistic operating conditions.

## Figures and Tables

**Table 1:** Material properties of the FGM constituents.

Material Properties	Ceramic	Metal
*μ*	0.3	0.3
*ρ* (kg/m^3^)	3800	7800
*E* (GPa)	380	210

**Table 2:** Comparison of the first 6 orders of frequency of the shaft–disk system with different power-law indices.

*p*	Method	Mode No.					
		1	2	3	4	5	6
0	Ref. [38]	133.60	530.73	1101.78	1914.37	3008.35	5258.03
	Present	133.65	529.89	1103.54	1920.90	3021.59	5299.08
	Error (%)	0.0378	0.1594	0.1600	0.3412	0.4401	0.7808
0.6	Ref. [38]	134.93	534.19	1099.68	1907.39	3004.11	5231.98
	Present	134.98	533.14	1100.21	1911.90	3013.32	5265.88
	Error (%)	0.0368	0.1962	0.0483	0.2365	0.3067	0.6480
1	Ref. [38]	135.69	536.04	1098.22	1903.15	3001.64	5216.73
	Present	135.74	534.68	1099.28	1905.65	3007.75	5243.96
	Error (%)	0.0361	0.2539	0.0964	0.1310	0.2035	0.5219
∞	Ref. [38]	147.85	514.40	974.73	1774.77	2923.61	4724.21
	Present	147.87	513.24	974.95	1770.57	2904.28	4688.20
	Error (%)	0.0174	0.2258	0.0226	0.2368	0.6611	0.7624

**Table 3:** Comparison of the first 9 orders of frequency for the system.

Method	Mode No.								
	1	2	3	4	5	6	7	8	9
FEM	142.36	142.36	524.44	524.44	531.77	716.63	716.63	839.09	839.09
Present	142.63	142.63	526.55	526.55	545.58	719.46	719.46	847.78	847.78
Error (%)	0.1862	0.1862	0.4028	0.4028	2.5977	0.3954	0.3954	1.0359	1.0359

**Table 4:** Comparison of the first 8 orders of frequency of the system at different rotational speeds.

Mode No.	Φ = 100 rad/s (955 rpm)			Φ = 200 rad/s (1910 rpm)			Φ = 300 rad/s (2865 rpm)		
	FEM	Present	Error (%)	FEM	Present	Error (%)	FEM	Present	Error (%)
1	129.00	129.15	−0.1163	115.35	115.11	0.2081	101.44	100.51	0.9168
	155.44	155.55	−0.0708	168.24	167.92	0.1902	180.73	179.71	0.5644
2	513.41	515.64	−0.4344	501.94	504.26	−0.4622	490.07	492.43	−0.4816
	535.00	536.97	−0.3682	545.05	546.87	−0.3339	554.57	556.25	−0.3029
3	531.53	550.18	−3.5087	530.82	532.87	−0.3862	529.64	543.51	−2.6188
4	711.52	714.40	−0.4048	706.71	709.74	−0.4287	702.19	705.45	−0.4643
	722.07	724.95	−0.3989	727.87	730.88	−0.4135	734.03	737.25	−0.4387
5	835.68	844.48	−1.0530	832.49	841.45	−1.0763	829.48	838.69	−1.1103
	842.72	851.37	−1.0264	846.59	855.25	−1.0229	850.73	859.44	−1.0238
6	1036.50	1036.19	0.0299	1036.50	1036.12	0.0367	1036.50	1035.99	0.0492
7	1089.90	1089.48	0.0385	1089.90	1089.46	0.0404	1089.90	1089.44	0.0422
8	1378.40	1377.67	0.0527	1378.20	1377.48	0.0522	1378.00	1377.28	0.0522
	1378.40	1377.67	0.0527	1378.20	1377.48	0.0522	1378.00	1377.28	0.0522

## Data Availability

The original contributions presented in this study are included in the article. Further inquiries can be directed to the corresponding author.

## Abbreviations

The following abbreviations are used in this manuscript:

FEM	Finite element method
FG	Functionally graded
FGM	Functionally graded material
FSDT	First-order shear deformation theory
